# Patient-reported outcome following an acetabular fracture: an observational study of 385 patients from the Swedish Fracture Register

**DOI:** 10.2340/17453674.2024.42414

**Published:** 2024-11-28

**Authors:** Madelene ALBREKTSSON, Michael MÖLLER, Mikael SUNDFELDT, David WENNERGREN, Olof WOLF, Carl BERGDAHL

**Affiliations:** 1 Department of Orthopaedics, Institute of Clinical Sciences, Sahlgrenska Academy, University of Gothenburg; 2 Department of Orthopaedics, Sahlgrenska University Hospital, Gothenburg/Mölndal; 3 Department of Orthopaedics and Hand Surgery, Uppsala University Hospital, Uppsala, Sweden

## Abstract

**Background and purpose:**

The primary aim of this study was to assess the patient’s self-reported change in health 1 year after sustaining an acetabular fracture. The secondary objective was to examine differences in patient-reported outcomes (PROMs) based on sex, age groups, injury mechanisms, type of fracture, and treatment.

**Methods:**

Data was collected from the Swedish Fracture Register (SFR) for patients with acetabular fractures sustained between 2014 and 2021. Patients with additional fractures at the time of injury or during the following 18 months, periprosthetic fractures, or pediatric fractures were excluded. The PROM used was the Short Musculoskeletal Function Assessment (SMFA) wherein the subindices of bother, dysfunction, and mobility were analyzed with a higher score indicating worse outcome. The differences in SMFA and in subindices between the score 1 year after fracture and preinjury (recall) were analyzed.

**Results:**

Of the 385 included patients with complete PROMs, there was no significant difference in changes in SMFA score between the sexes. Surgically treated patients had significantly higher scores 1 year post-injury compared with non-surgically treated patients with bother index 18.3 (95% confidence [CI] 14.0–22.6) vs 7.2 (CI 4.7–9.8), dysfunction index 15.8 (CI 12.7–18.9) vs 7.0 (CI 5.0–9.0), and mobility index 21.6 (CI 17.9–25.2) vs 9.2 (CI 6.9–11.5).

**Conclusion:**

Most patients sustaining an acetabular fracture experience a decline in their functional abilities 1 year after the injury compared with before the injury. Younger patients with high-energy injuries and complex fracture types, which typically require surgical intervention, experience the most unfavorable outcomes. The large group of non-surgically treated patients reported minimal functional changes, likely attributable to selection bias.

The incidence of acetabular fractures is increasing, especially among older adults in the Western world [1,2]. Earlier treatment was almost exclusively non-surgical, but treatment strategies change continuously, and surgical treatment is becoming more frequent [3]. However, 75% of acetabular fracture patients are still treated non-surgically [4].

Studies of functional outcome following an acetabular fracture have focused on surgically treated patients at single centers and there is a lack of research on patient-reported outcomes following non-surgical treatment [5-12]. Moreover, an array of scoring systems, such as the system described by Merle d’Aubigné, the SF-36 (36-Item Short Form Health Survey), and the Harris Hip Score (HHS), have been used as outcome measures. Due to the evolving patient spectrum and advancements in treatment options, it is essential to assess treatment outcomes consistently for all patients with acetabular fractures.

The primary aim of this study was to assess outcomes in patients sustaining different types of acetabular fractures, both surgically and non-surgically treated, using patient-reported outcome measures (PROMs) from the Swedish Fracture Register (SFR). We also examined variations in outcome scores across sex, age categories, causes of injury, fracture types, and treatment groups.

## Methods

### Setting

This is an observational register study of prospectively collected data from the SFR. It is reported according to STROBE reporting guidelines.

The SFR is a national quality register that started in 2011 and reached 100% coverage among orthopedic trauma departments in Sweden in 2020 [13]. Completeness for acetabular and pelvic fractures was 45% in 2021 when compared with the National Patient Register (NPR) [14]. However, the NPR overestimates the number of fractures because of multiple registrations of the same fracture and completeness in the SFR is therefore underestimated [15]. Both in- and outpatients are registered in the SFR by the treating orthopedic surgeon regardless of treatment modality. The classification of acetabular fractures in the SFR has previously been validated showing moderate agreement with the established gold standard [16]. PROM questionnaires evaluate functional outcomes and the patient’s health status.

### Selection criteria and study variables

Data was extracted on all patients aged ≥ 16 years with an acetabular fracture registered in the SFR between January 1, 2014 and January 1, 2022. Patients with a concomitant fracture at the time of injury or any other fracture within 18 months after the acetabular fracture were excluded prior to data collection to avoid other fractures affecting PROMs. After data retrieval, patients with incomplete PROM responses, periprosthetic fractures, or pediatric fractures (open physes) were excluded from the final analyses. Demographic data for patients with incomplete PROMs was used to compare responders with non-responders.

Information on the patient’s age, sex, the fracture classification, injury energy level, treatment type (surgical or non-surgical), and PROM scores was collected from the SFR for analysis. Patients were divided into 2 age groups for subgroup analysis: > 70 and ≤ 70 years.

### Fracture classification

Fractures were classified by the treating physician according to the AO/OTA classification, which contains the same classification groups as described by Judet and Letournel [5,17,18].

### Outcome

The SMFA (Short Musculoskeletal Function Assessment), a 46-item tool, was employed as the PROM questionnaire. The SMFA is a validated tool to measure a broad range of musculoskeletal injuries, although not specifically validated for acetabular fracture patients [19]. The questionnaire comprises 2 subindices: the dysfunction and bother indices [20]. The dysfunction index is subdivided into 4 categories: daily activities, emotional status, function of the arm and hand, and mobility. The dysfunction index describes the amount of difficulty the patient experiences when performing a defined task. The bother index focuses on how much the patient is bothered by their injury in different broad functional areas. Each item in the SMFA questionnaire has 5 response options.

After index calculation the score ranges from 0 to 100. The higher the score the poorer the function. Pre-injury scores (PROM 0) were collected using recall technique, i.e., asking the patients to describe their preinjury function, within 3 weeks of injury. Scores 1 year post-fracture (PROM 1) were collected only from patients who had answered the PROM 0. A change in health was assessed by subtracting the PROM 0 score from the PROM 1 score. The larger the difference, the greater reported impairment. The present study analyzed the bother index and the dysfunction index. The mobility subcategory of the dysfunction index was deemed most relevant to patients with an acetabular fracture and was therefore also presented separately.

### Statistics

The change in health status between PROM 0 and PROM 1 was calculated on an individual level for each PROM variable. On a group level, mean values were used to describe the change in the health and quality of life 1 year after injury. Variables were presented as numbers, proportions or median, and interquartile range (IQR), excluding missing values.

Comparisons of demographic data between responders and non-responders were analyzed and presented as differences in proportions with 95% confidence intervals (CIs). Differences in median age were analyzed using independent samples Hodges–Lehman estimate. The findings in the current study can be considered valid under the assumption that the missing PROM data (non-responders’ data) is missing completely at random (MCAR) [21].

Statistical analyses were conducted using SPSS Statistics (version 29, IBM Corp, Armonk, NY, USA).

### Ethics, registration, data sharing plan, use of AI, funding, and disclosures

Ethics approval was obtained from the Swedish Ethical Review Authority (registration number 2020-03775 approved September 25, 2020 and 2023-01499-02 approved March 27, 2023). The SFR data in this study is not publicly available due to Swedish legislation on public access and secrecy. Individuals interested in this dataset can apply to retrieve data from the Center of Registers, Västra Götaland, Sweden after ethics approval from the Swedish Ethical Review Authority. There was no use of artificial intelligence tools (AI) in conducting this study or in writing this manuscript. None of the authors have any conflicts of interest or funding to declare. Complete disclosure of interest forms according to ICMJE are available on the article page, doi: 10.2340/17453674.2024.42414

## Results

**Figure 1:**
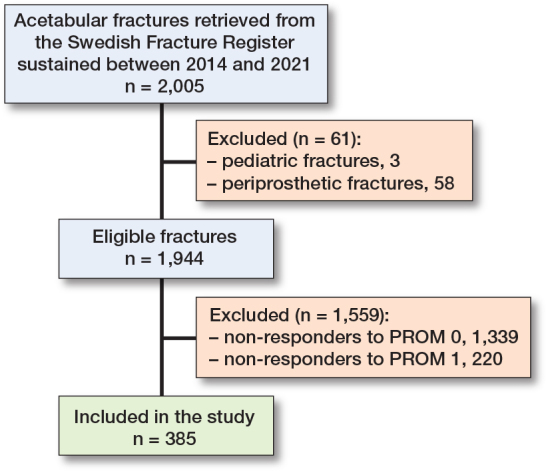
Flowchart of patients included in the study. PROM = patient-reported outcome measures, 0 = before injury, 1 = at 1 year.

Of 1,944 eligible fractures, 1,339 were excluded due to missing PROM 0 and 220 for missing PROM 1 (Figure 1). The responding study cohort of 385 patients did not differ significantly from non-responders (n = 1,557) in the distribution of sex or type of fracture (Table 1). However, the median age in the responding group was significantly lower than in the non-responders (71 years vs 79 years, respectively) and there was a larger proportion of high-energy injuries and surgical treatment among responders.

**Table 1:** Demographics of the responders and non-responders. Values are count (%) unless otherwise specified and difference with 95% confidence intervals (CI)

Characteristic	Overall (n = 1,944)	Responders (n = 385)	Non-responders (n = 1,559)	Difference (CI)^a^
Male sex	1,247 (64)	256 (66)	991 (64)	2.9 (–2.4 to 8.1)
Median age (IQR)				
All	77 (65–86)	71 (60–79)	79 (66–87)	7 (6 to 9)
Male	74 (62–84)	70.5 (60–77)	76 (63–85)	
Female	83 (71–89)	74 (58–84)	84 (74–90)	
Type of energy				
High energy	288 (15)	87 (23)	201 (13)	11 (5.6 to 15)
Low energy	1,298 (67)	229 (59)	1,069 (69)	12 (6.3 to 17)
Unknown	311 (16)	62 (16)	249 (16)	
Not applicable	47 (2.4)	7 (1.8)	40 (2.6)	
Type of fracture				
Elementary fracture types				
Posterior wall	237 (12)	52 (14)	185 (12)	1.6 (–2.0 to 5.6)
Posterior column	162 (8.3)	30 (7.8)	132 (8.5)	0.7 (–2.5 to 3.5)
Anterior wall	400 (21)	78 (20)	322 (21)	0.4 (–4.2 to 4.8)
Anterior column	191 (10)	34 (8.8)	157 (10)	1.2 (–2.1 to 4.3)
Pure transverse	163 (8.4)	33 (8.6)	130 (8.3)	0.2 (–3.5 to 2.7)
Associated fracture types				
Posterior column and posterior wall	113 (5.8)	28 (7.3)	85 (5.5)	1.8 (–0.9 to 4.8)
Transverse and posterior wall	73 (3.8)	16 (4.2)	57 (3.7)	0.5 (–1.6 to 2.9)
T-shaped	144 (7.4)	24 (6.2)	120 (7.7)	1.5 (–1.5 to 4.1)
Anterior and posterior hemitransverse	113 (5.8)	23 (6.0)	90 (5.8)	0.2 (–2.3 to 3.0)
Both column	149 (7.7)	36 (9.4)	113 (7.2)	2.1 (–0.9 to 5.5)
Unclassified	199 (10)	31 (8.1)	168 (11)	2.7 (–0.6 to 5.7)
Primary treatment				
Surgical	426 (22)	121 (31)	305 (20)	13 (7.6 to 18)
Non-surgical	1,416 (73)	241 (63)	1,175 (75)	
Unknown	102 (5.2)	23 (6.0)	79 (5.1)	

a Difference between responders and non-responders is accounted for in percentage points, except median age, which is in years. IQR = interquartile range.

### PROM change

Overall, the study cohort reported impaired function 1 year post-fracture. Mean increases in SMFA were between 10.2 (CI 8.5–11.9) and 13.7 (CI 11.7–15.7) for all 3 SMFA indices (Table 2), with the largest increase in the mobility index. Likewise, the mobility index had the largest increase when subgroups (i.e., sex, age, injury type, and treatment modality) were analyzed (Tables 2–4).

**Table 2:** Change in patient-reported function stratified by sex and age group, 1 year after sustaining an acetabular fracture compared with 1 week before the injury. Values are count and mean change in Short Musculoskeletal Function Assessment subindices with 95% confidence intervals (CI)

Patient group	Bother		Dysfunction		Mobility	
	n	mean (CI)	n	mean (CI)	n	mean (CI)
All	347	11.3 (9.1–13.5)	382	10.2 (8.5–11.9)	382	13.7 (11.7–15.7)
Male	237	11.8 (9.2–14.4)	253	9.9 (7.9–12.0)	253	13.2 (10.8–15.5)
Female	110	10.1 (5.9–14.4)	129	10.8 (7.8–13.8)	129	14.7 (11.1–18.3)
≤ 70 years	172	13.8 (10.4–17.1)	182	11.4 (9.0–13.9)	182	15.5 (12.5–18.4)
> 70 years	175	8.8 (5.9–11.7)	200	9.1 (6.8–11.5)	200	12.0 (9.3–14.8)

**Table 3:** Change in patient-reported function stratified by energy level of the injury, 1 year after sustaining an acetabular fracture compared with 1 week before the injury. Values are count and mean change in Short Musculoskeletal Function Assessment subindices with 95% confidence intervals (CI)

Energy level	Bother		Dysfunction		Mobility	
	n	mean (CI)	n	mean (CI)	n	mean (CI)
High	84	13.7 (8.3–19.1)	86	13.3 (9.7–16.9)	86	17.8 (13.5–22.0)
Low	200	10.6 (8.1–13.1)	228	9.8 (7.7–12.0)	228	12.5 (10.0–15.1)

**Table 4:** Change in patient-reported function stratified by treatment and age group, 1 year after sustaining an acetabular fracture compared with 1 week before the injury. Values are count and mean change in Short Musculoskeletal Function Assessment subindices with 95% confidence intervals (CI)

Treatment Age	Bother		Dysfunction		Mobility	
	n	mean (CI)	n	mean (CI)	n	mean (CI)
Surgical						
All	108	18.3 (14.0–22.6)	120	15.8 (12.7–18.9)	120	21.6 (17.9–25.2)
≤ 70 years	72	20.5 (14.6–26.3)	78	17.9 (13.8–22.1)	78	24.1 (19.4–28.9)
> 70 years	36	13.9 (8.3–19.6)	42	11.8 (7.4–16.2)	42	16.8 (11.4–22.2)
Non-surgical						
All	218	7.2 (4.7–9.8)	239	7.0 (5.0–9.0)	239	9.2 (6.9–11.5)
≤ 70 years	91	6.7 (3.2–10.2)	95	5.0 (2.6–7.4)	95	6.9 (4.1–9.8)
> 70 years	127	7.6 (4.1–11.2)	144	8.4 (5.4–11.3)	144	10.7 (7.4–14.1)

### Sex and age

Although not statistically significant, younger patients (≤ 70 years) reported a larger increase in all subindices than the older group (> 70 years) (Table 2). The mean change in SMFA score was similar between the sexes.

### Injury mechanism and primary treatment

Patients with fractures due to high-energy trauma reported a larger, but not statistically significant, change in SMFA for all subindices than patients with fractures following low-energy trauma (Table 3). Surgically treated patients had significantly larger change in SMFA compared with non-surgically treated patients (Table 4). Patients treated non-surgically had a mean change in SMFA of 7.0–9.2, whereas for surgically treated patients this was 15.8–21.6. In the surgically treated group, there was a tendency for younger patients (≤ 70 years) to report worse outcome scores compared with older patients (> 70 years). This finding could not be statistically established but a corresponding age-related difference in reported outcome could not be found in the non-surgically treated group.

### Fracture type

**Figure 2:**
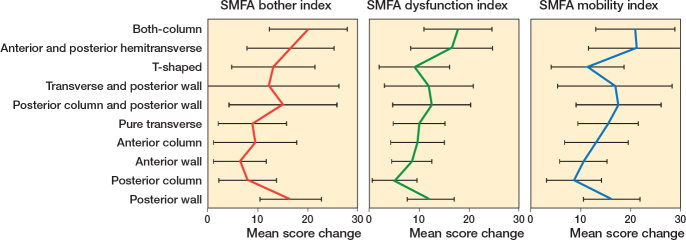
Differences in patient-reported function stratified by fracture type, 1 year after sustaining an acetabular fracture compared with 1 week before the injury. A positive value denotes a decrease in function, a negative an improvement. Data is mean with 95% confidence intervals. SMFA = Short Musculoskeletal Function Assessment.

No statistically significant differences were found between fracture types. However, there were numerical differences. The fracture types with the largest increase in mean SMFA were the both-column fractures and the anterior and posterior hemitransverse fractures (scores 16.5–21.2; Figure 2). Patients with fractures involving the posterior wall of the acetabulum (posterior wall fractures, posterior column and posterior wall, and transverse and posterior wall fractures) also had a large increase in SMFA scores. Patients who sustained fractures of the anterior wall and posterior column reported the least impact on functional ability 1 year after the injury.

## Discussion

The study aimed to present patient-reported changes in health 1 year after surgically and non-surgically treated acetabular fractures. We have shown that acetabular fractures cause functional impairment in all patient groups 1 year post-fracture, regardless of fracture pattern or the treatment approach (surgical or non-surgical). Although not statistically significant, our results indicated more severe functional impairment in younger patients (≤ 70 years) and those who suffered high-energy trauma. More complex fracture patterns and fractures involving the posterior wall had the worst functional outcomes of all fracture types.

The overall result for the entire cohort demonstrates a reduction in function 1 year after sustaining an acetabular fracture. Due to differences in measurement instruments and the almost unique inclusion of non-surgically treated patients in the current study, a direct comparison with the result from other studies is difficult. However, our results align with findings from Walley et al. on functional impairment following acetabular fractures. They primarily included older adults not returning to their previous level of ambulation following acetabular fractures, regardless of treatment [22]. Although not using patient-reported scores, Baker et al. also described a significant reduction in mobility and independence after 1 year of non-surgical treatment for complex acetabular fractures among an older population considered too frail for surgical treatment [23]. The current study adds to this information by including not only non-surgical treatment due to frailty or age but also a large group of patients whose treatment of choice is non-surgical.

There was no significant statistical difference in patient-reported function between the sexes. The younger population (≤ 70 years) demonstrated greater impairment. This finding presents a contradiction to previous findings, which indicated that advanced age was a prognostic factor for unfavorable outcomes after acetabular fracture surgery with open reduction and internal fixation (ORIF) [6,9]. The present results may have several explanations. One could be that younger patients exhibit greater expectations regarding limb function, thus perceiving even a minor impairment as a substantial decline in functionality. Another plausible explanation is that acetabular fractures in patients ≤ 70 years are more often the result of high-energy trauma mechanisms, which may cause a more serious injury than fractures due to low-energy traumas [4]. In our study, high-energy trauma was associated with worse impairment compared with patients with low-energy trauma. Additionally, there is a current trend to prioritize primary hip arthroplasty over ORIF as the preferred treatment for older patients with complex fractures, as indicated by reported encouraging results [24,25]. This change in treatment practice might partly explain why older adults in our study reported comparatively reduced functional impairment.

We showed a statistical difference between surgically and non-surgically treated patients regarding change in SMFA 1 year post-injury. However, the study’s observational nature hinders a direct comparison between these patients and it seems plausible that the unfavorable outcome in surgically treated patients is attributable to selection bias. High-demand patients and fractures with greater displacement are more often treated surgically, leading to worse functional outcomes in the surgically treated cohort compared with the non-surgical cohort.

Our result of a relatively modest decline in function 1 year post-fracture following non-surgical treatment agrees with a Norwegian study reporting good or excellent outcome scores for almost 90% of non-surgically treated patients with minimally displaced acetabular fractures [26]. Treatment decisions are often influenced by a combination of patient demand and fracture characteristics, such as degree of displacement. The difference in outcome for surgically and non-surgically treated patients in our study and the relatively small decreases in function 1 year after injury for the non-surgically treated patients could indicate that the current indications for surgery and patient selection are reasonable.

Consistent with previous research, the current study indicates that fractures involving the posterior wall have greater impairment than fractures without posterior wall engagement [12,27]. However, the worst outcomes were reported by patients sustaining both column and anterior and posterior hemitransverse fractures. These more complex fracture types will have a greater impact on causing severe disabilities. This assumption is in line with reports showing that patients with associated fracture patterns tend to experience more unfavorable outcomes [10].

### Limitations

The low response rate is an obvious limitation. Even though this is the largest study on patient-reported outcome following acetabular fractures, few statistically significant differences were detected. Moreover, there were some baseline differences between responders and non-responders. A larger proportion of the responders underwent operative treatment and suffered a higher number of high-energy injuries compared with the non-responders. Although this might skew the results, Juto et al. showed in their validation study on responders and non-responders in the SFR that responders to PROM 1 generally have more problems than non-responders but that the difference disappeared after case-control matching [28]. Comorbidity may differ between responders and non-responders. The clinical relevance of a change in SMFA scores or a change considered important by the patients has, to our knowledge, not been studied for the Swedish version.

### Strengths

The register-based design of the study allowed for the inclusion of a comparatively large cohort of patients. The study design also made possible the inclusion of all fracture types and both surgical and non-surgical treatment. As a result of the nationwide coverage of the SFR, all hospitals of varying sizes in Sweden took part, although most acetabular surgeries are centralized to university hospitals [13]. The SMFA, including the Swedish translation, has been validated and tested for reliability and responsiveness and has proven to be a good assessment tool for patients with various musculoskeletal disorders [19].

### Conclusion

Most patients with an acetabular fracture encounter modest functional impairment 1 year post-injury. The greatest numerical decline in functional outcome was found among patients ≤ 70 years, patients with high-energy injuries or complex fracture types, and surgically treated patients. In perspective, extensive comparative studies are warranted to refine the current treatment concepts of acetabular fractures.

## Notes

MA: data curation, formal analysis, methodology, visualization, writing—original draft. MM: conceptualization, funding acquisition, project administration, methodology, supervision, writing—review & editing. MS: conceptualization, methodology, supervision, writing—review & editing. CB, DW, OW: methodology, supervision, writing—review & editing. 

Handling co-editors: Marianne Westberg and Robin Christensen

*Acta* thanks Jeppe Barckman and Morten Schultz Larsen for help with peer review of this manuscript.
